# Sphingosine kinase 1 regulates HMGB1 translocation by directly interacting with calcium/calmodulin protein kinase II-δ in sepsis-associated liver injury

**DOI:** 10.1038/s41419-020-03255-6

**Published:** 2020-12-06

**Authors:** Tao Tian, Danhua Yao, Lei Zheng, Zhiyuan Zhou, Yantao Duan, Bin Liu, Pengfei Wang, Yousheng Li

**Affiliations:** Department of General Surgery, Shanghai Ninth People’s Hospital, Shanghai JiaoTong University School of Medicine, Shanghai, People’s Republic of China

**Keywords:** Hepatotoxicity, Sepsis

## Abstract

Previously, we confirmed that sphingosine kinase 1 (SphK1) inhibition improves sepsis-associated liver injury. High-mobility group box 1 (HMGB1) translocation participates in the development of acute liver failure. However, little information is available on the association between SphK1 and HMGB1 translocation during sepsis-associated liver injury. In the present study, we aimed to explore the effect of SphK1 inhibition on HMGB1 translocation and the underlying mechanism during sepsis-associated liver injury. Primary Kupffer cells and hepatocytes were isolated from SD rats. The rat model of sepsis-associated liver damage was induced by intraperitoneal injection with lipopolysaccharide (LPS). We confirmed that Kupffer cells were the cells primarily secreting HMGB1 in the liver after LPS stimulation. LPS-mediated HMGB1 expression, intracellular translocation, and acetylation were dramatically decreased by SphK1 inhibition. Nuclear histone deacetyltransferase 4 (HDAC4) translocation and E1A-associated protein p300 (p300) expression regulating the acetylation of HMGB1 were also suppressed by SphK1 inhibition. HDAC4 intracellular translocation has been reported to be controlled by the phosphorylation of HDAC4. The phosphorylation of HDAC4 is modulated by CaMKII-δ. However, these changes were completely blocked by SphK1 inhibition. Additionally, by performing coimmunoprecipitation and pull-down assays, we revealed that SphK1 can directly interact with CaMKII-δ. The colocalization of SphK1 and CaMKII-δ was verified in human liver tissues with sepsis-associated liver injury. In conclusion, SphK1 inhibition diminishes HMGB1 intracellular translocation in sepsis-associated liver injury. The mechanism is associated with the direct interaction of SphK1 and CaMKII-δ.

## Introduction

Sepsis, a life-threatening disease caused by infection, is characterized by high mortality and treatment difficulties^1^. Multiple organ dysfunction may be induced by sepsis. Liver injury is one of the common complications for sepsis patients^2^. The occurrence of sepsis-associated liver damage indicates a poor outcome and high hospital mortality^3,4^. Therefore, it is crucial to explore the pathogenesis of sepsis-associated liver injury.

High-mobility group box 1 (HMGB1) is a late mediator of lethal systemic inflammation. HMGB1 is normally located in the nucleus, and can be translocated from the nucleus to the cytoplasm and released extracellularly by specific stimulators. HMGB1 intracellular translocation was observed in patients with acute liver failure (ALF)^5^. Neutralization of HMGB1 with antibody significantly improved liver damage and survival in a rat model of ALF induced by d-galactosamine^6^. These studies have demonstrated that HMGB1 may participate in the pathogenesis of sepsis-associated liver damage^7^.

Sphingosine kinase 1 (SphK1), an intracellular signaling enzyme, plays a key role in inflammatory responses^8–11^. The expression of SphK1 was increased in lipopolysaccharide (LPS)-stimulated macrophages^8^ and microglia^9^. Elevated SphK1 expression and enzyme activity were observed in severe acute pancreatitis patients, and were positively related to disease severity^10^. We previously reported that SphK1 expression was elevated in LPS+ d-galactosamine-induced liver failure^11^. Furthermore, SphK1 inhibition ameliorated sepsis-associated liver damage^11^. However, whether SphK1 may regulate HMGB1 translocation to mediate the development of sepsis-associated liver injury remains unknown.

In this study, we aimed to investigate the effect of SphK1 inhibition on HMGB1 translocation and the underlying mechanism of sepsis-associated liver injury.

## Materials and methods

### Reagents

LPS (*Escherichia coli* 0111: B4), SKI-5C, K6PC-5, and glycyrrhizin were purchased from Sigma-Aldrich (Shanghai, China). Gadolinium chloride (GdCl3) was obtained from Absin (Shanghai, China). Collagenase IV, protein interaction pull-down kit, anti-CD68, anti-phospho-HDAC4, anti-calcium/calmodulin protein kinase II-δ (CaMKII-δ), and anti-phospho-CaMKII-δ were obtained from Thermo Scientific (Shanghai, China). Anti-SphK1 and anti-HMGB1 (ChIP Grade) were obtained from Abcam (Shanghai, China). Anti-HMGB1, anti-GAPDH, anti-Lamin B1, anti-histone H3, anti-E1A-associated protein p300 (p300), anti-CREB-binding protein (CBP), anti-p300/CBP-associated factor (PCAF), anti-histone deacetylase (HDAC) 1, anti-HDAC4, and anti-acetylated-lysine were purchased from Cell Signaling Technology (Shanghai, China). Anti-HMGB1 (Acetyl-Lys12) was obtained from Aviva Systems Biology (San Diego, CA, USA). Anti-HA and anti-His were purchased from Zoonbio Technology (Nanjing, China). The manufacturers of all the antibodies used in this study are listed in Supplementary Table 1.

### Animals and treatment

All experimental procedures were consistent with the animal ethics of Shanghai Ninth People’s Hospital. Male Sprague Dawley (SD) rats (6–8 weeks) were housed under general conditions. The rat model of sepsis-associated liver damage was induced by intraperitoneal injection with LPS (8 mg/kg)^12^. To specifically deplete Kupffer cells, GdCl3 (20 mg/kg) was given intraperitoneally 24 h before LPS injection^12^.

### Human liver samples

Human liver tissues from sepsis-associated liver injury were obtained from Shanghai Ninth People’s Hospital. The use of these samples was approved by the ethics committee of Shanghai Ninth People’s Hospital. Written informed consent was obtained from all patients. The characteristics of the patients are listed in Supplementary Table 2.

### Cell isolation, culture, and treatment

The protocols for isolating primary Kupffer cells and hepatocytes from the SD rats are shown in the Supplementary Information.

Primary rat Kupffer cells were cultured in Dulbecco’s Modified Eagle Medium (DMEM) supplemented with 10% fetal bovine serum (FBS), 100 U/ml penicillin, and 100 μg/ml streptomycin. Primary hepatocytes were seeded on plates precoated with rat tail collagen and cultured in William’s E Medium supplemented with 10% FBS, 100 U/ml penicillin, and 100 μg/ml streptomycin. RAW264.7 cells were used to investigate the underlying mechanism.

The cells were stimulated with LPS (1 μg/ml) for 16 h. K6PC-5 (100 μM), a selective SphK1 activator, was added 24 h before the LPS treatment.

### SphK1 inhibition

SKI-5C is a specific SphK1 inhibitor. The compound was first developed by Wong et al. in 2009 (ref. ^13^). Compared with *N*,*N*-dimethylsphingosine (DMS), SKI-5C showed potential specificity for SphK1 inhibition and less toxicity^13^. It has been widely used in SphK1 inhibition^11,14,15^. SKI-5C (2 mg/kg) was administered intraperitoneally 30 min before the LPS injection^11,13^. Kupffer cells were treated with SKI-5C (10 μM) 24 h before the LPS treatment^14^.

### HMGB1 inhibition

Glycyrrhizin is a well-known HMGB1 inhibitor. Glycyrrhizin has been shown to be able to directly bind to HMGB1 (ref. ^16^), suppress HMGB1 expression, and inhibit HMGB1 activities^17,18^. Kupffer cells were treated with glycyrrhizin (100 μM) 24 h before the LPS treatment.

### Separation of cytoplasmic and nuclear extracts

Cytoplasmic and nuclear extracts were separated by cell fractionation kit (Cell Signaling Technology, Shanghai, China) according to the manufacturer’s instructions (see Supplementary Information for detailed descriptions).

### Immunoprecipitation, co-immunoprecipitation, and immunoblotting

Cell lysates were prepared with RIPA buffer. Cell lysate preclearing was recommended before the immunoprecipitation (IP) step. Then primary antibodies were added to the cell lysates. After incubation overnight at 4 °C, prewashed protein A/G magnetic beads were added to the immunocomplex. The samples were heated to 95–100 °C following a washing step. Then, the samples were analyzed by immunoblotting as described previously^11^ (see Supplementary Information for detailed descriptions).

### Pull-down assay

The HA–SphK1 fusion protein was cloned into a pGEX4T-1 vector, and the His-CaMKII-δ fusion protein was cloned into a pCzn1 vector and transformed into BL21 *E. coli*. The fusion proteins were purified using nickel column purification. HA-tagged SphK1 and HA polypeptides were added to HA resin separately, and rotated for 4 h at 4 °C. Then His-tagged CaMKII-δ was added to the mixture. The mixture was rotated overnight at 4 °C. After elution, the protein samples were separated and detected with immunoblotting (see Supplementary Information for detailed descriptions).

### Enzyme activity assay

Nuclear extracts were prepared with a cytoplasmic and nuclear fractionation kit (Invent, Beijing, China). Specifically, sanction is an effective method to separate nuclear extracts. Histone acetyltransferases (HATs) and histone deacetyltransferases (HDACs) activity in the nuclear extracts were detected by colorimetric assay kits (BioVision, Milpitas, USA.). CaMKII activity was measured using a commercial assay kit (Genmed, Shanghai, China). The detailed method is presented in the Supplementary Information.

### ELISA

HMGB1 in serum, cell lysates, and supernatants was measured with an ELISA kit (IBL International). For a detailed method, see the Supplementary Information.

### Immunofluorescence

The samples were fixed with 4% formaldehyde. After blocking, primary antibodies were added to the samples and incubated overnight at 4 °C. Then the samples were incubated with a fluorochrome-conjugated secondary antibody. Images were obtained with a Nikon fluorescence microscope. The detailed protocol is included in the Supplementary Information.

### Statistical analysis

Data are expressed as the means ± standard deviation. Two sets of data were compared with Student’s *t*-test. Differences between the groups were analyzed with a one-way analysis of variance (ANOVA) followed by Bonferroni post hoc test with SPSS 17.0 software. A square-root transformation was performed when the variances were unequal. A value of *P* < 0.05 was statistically significant.

## Results

### Kupffer cells were the primary cells secreting HMGB1 in the liver

**Fig. 1:**
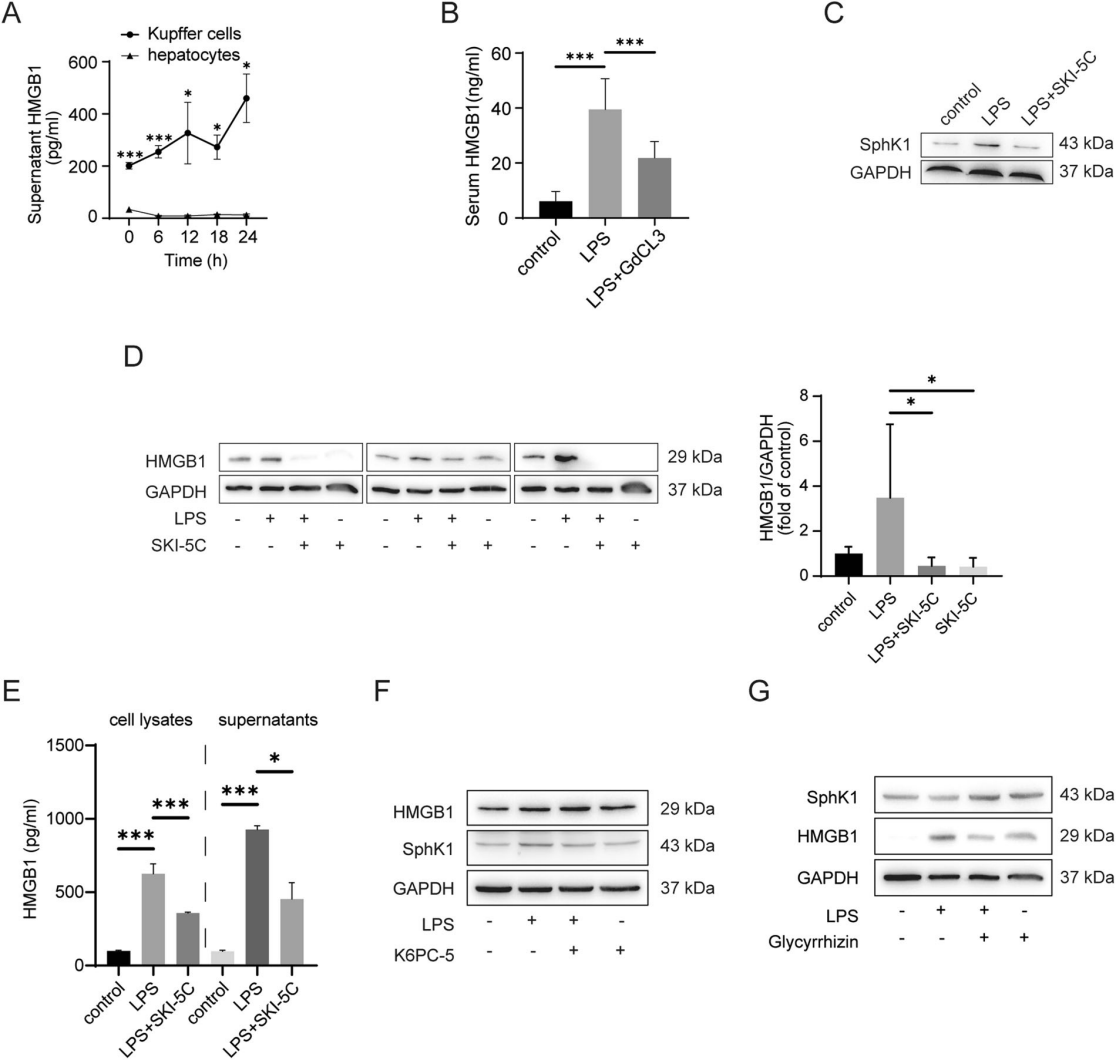
Kupffer cells are the major cells secreting HMGB1 in the liver and HMGB1 expression can be attenuated by SphK1 inhibition in Kupffer cells. **A** ELISA analysis of supernatant HMGB1 in primary rat Kupffer cells and hepatocytes after LPS (1 μg/ml) stimulation. **B** Rats received intraperitoneal injection of LPS (8 mg/kg) in the absence or presence of GdCl3 (20 mg/kg for 24 h before the LPS injection) by intraperitoneal injection. Twelve hours after the injection, serum HMGB1 was measured by ELISA (*n* = 8). **C** SKI-5C inhibited SphK1 expression, as indicated by immunoblot assay of Kupffer cells. **D** Kupffer cells were pretreated with SKI-5C (10 μM for 24 h) and then stimulated with LPS (1 μg/ml for 16 h). HMGB1 expression was measured by immunoblotting. The data represent three independent experiments. **E** The same as **D**, except that HMGB1 levels in the cell lysates and supernatants were measured by ELISA. **F** K6PC-5 is a selective SphK1 activator. Kupffer cells were pretreated with K6PC-5 (100 μM for 24 h) and then stimulated with LPS (1 μg/ml for 16 h). HMGB1 expression was measured by immunoblotting. **G** Glycyrrhizin is a known HMGB1 inhibitor. Kupffer cells were pretreated with glycyrrhizin (100 μM for 24 h) and then stimulated with LPS (1 μg/ml for 16 h). SphK1 expression was measured by immunoblotting. Data are expressed as the means ± standard deviation. *P < 0.05, **P < 0.01, ***P < 0.001.

We explored whether hepatocytes or Kupffer cells were the major cells secreting HMGB1 during sepsis-associated liver damage. First, we isolated primary Kupffer cells and hepatocytes from SD rats (Fig. S1). Rat primary Kupffer cells and hepatocytes were stimulated by LPS for 16 h in vitro. The HMGB1 level in the Kupffer cells supernatant was significantly higher than that in the hepatocyte supernatant after the cells were treated with LPS in vitro (Fig. 1A). GdCl3, a known macrophage inhibitor, was used to investigate the influence of Kupffer cell depletion on HMGB1 secretion. As illustrated in Fig. 1B, serum HMGB1 levels were dramatically downregulated by GdCl3 in vivo (*P* < 0.001, compared with the LPS group).

### HMGB1 expression was attenuated by SphK1 inhibition in Kupffer cells

Since previous studies showed that the expression of HMGB1 and SphK1 is elevated in sepsis-associated liver damage, whether HMGB1 and SphK1 are related remains unknown. In our study, SphK1 was enhanced in LPS-stimulated Kupffer cells and inhibited by SKI-5C (Fig. 1C). The protein expression of HMGB1 was reduced after SKI-5C administration in vitro, as indicated by immunoblotting (Fig. 1D, *P* < 0.05). Furthermore, the HMGB1 levels in cell lysates and supernatants were also decreased by SKI-5C treatment (Fig. 1E, *P* < 0.001, compared with the LPS group). However, HMGB1 expression was not changed by a selective SphK1 activator in vitro (Fig. 1F). Glycyrrhizin inhibited HMGB1 expression and did not alter SphK1 expression in vitro (Fig. 1G).

### HMGB1 intracellular translocation and acetylation were suppressed by SphK1 inhibition

**Fig. 2:**
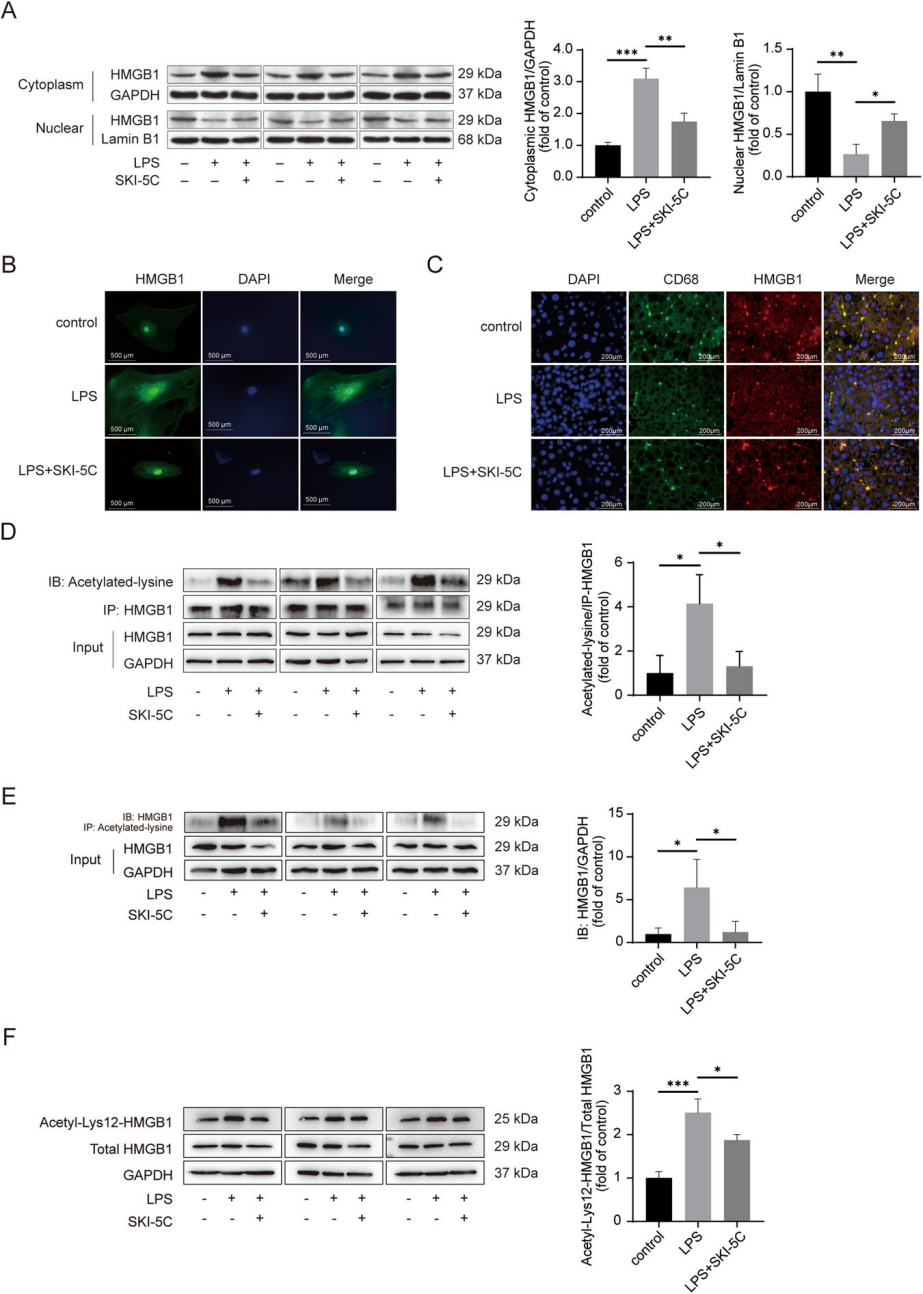
HMGB1 intracellular translocation and acetylation can be suppressed by SphK1 inhibition. **A** Kupffer cells were pretreated with SKI-5C (10 μM for 24 h) and then stimulated with LPS (1 μg/ml for 16 h). HMGB1 translocation from the nucleus to the cytoplasm was measured by immunoblotting. Data represent three independent experiments. **B** Kupffer cells were pretreated with SKI-5C (10 μM for 24 h) and then stimulated with LPS (1 μg/ml for 16 h). HMGB1 intracellular translocation was analyzed by immunofluorescence assay. **C** Rats received intraperitoneal injection of LPS (8 mg/kg) in the absence or presence of SKI-5C (2 mg/kg for 30 min before LPS injection) by intravenous injection. Twelve hours after the injection, intracellular HMGB1 translocation in CD68-marked Kupffer cells obtained from liver tissue was analyzed by immunofluorescence assay. **D** RAW264.7 cells were pretreated with SKI-5C (10 μM for 24 h) and then stimulated with LPS (1 μg/ml for 16 h). The acetylation of HMGB1 was measured by immunoprecipitation with an anti-HMGB1 antibody and immunoblotting with anti-acetylated-lysine antibody. The data represent three independent experiments. **E** The same as **D**, except that the acetylation of HMGB1 was measured by immunoprecipitating with anti-acetylated-lysine antibody and immunoblotting with anti-HMGB1 antibody. Data represent three independent experiments. **F** The same as **D**, except that the acetylation of HMGB1 was measured by immunoblotting with anti-acetyl-HMGB1 (Lys12) antibody. The data represent three independent experiments. Data are expressed as the means ± standard deviation. *P < 0.05, **P < 0.01, ***P < 0.001.

HMGB1 may be translocated from the nucleus to the cytoplasm when stimulated by LPS. We sought to determine whether SphK1 inhibition might influence HMGB1 translocation. Analyses of the separated cytoplasmic and nuclear extracts revealed that the HMGB1 intracellular shift was inhibited by SKI-5C in Kupffer cells (Fig. 2A). Immunofluorescence assays further confirmed that SKI-5C inhibited HMGB1 intracellular translocation in Kupffer cells (Fig. 2B). Similarly, HMGB1 translocation in the CD68-marked Kupffer cells from liver tissue was attenuated by SKI-5C in vivo (Fig. 2C). HMGB1 hyperacetylation is the primary mechanism for this intracellular shift^19^. Then we detected the influence of SphK1 inhibition on HMGB1 acetylation in vitro. First, we performed an IP assay using anti-HMGB1 antibody and an immunoblot assay with anti-acetylated-lysine antibody, and the results which indicated that HMGB1 acetylation was downregulated by SKI-5C (Fig. 2D). Then, we performed an co-immunoprecipitation (co-IP) with anti-acetylated-lysine antibody and an immunoblot assay with anti-HMGB1 antibody. The results show that SKI-5C decreased the levels of HMGB1 acetylation (Fig. 2E). Immunoblotting with anti-acetyl-HMGB1 (Lys12) antibody was also performed to verify the suppression of HMGB1 acetylation by SKI-5C (Fig. 2F).

### SphK1 inhibition suppressed p300 expression and HDAC4 translocation

**Fig. 3:**
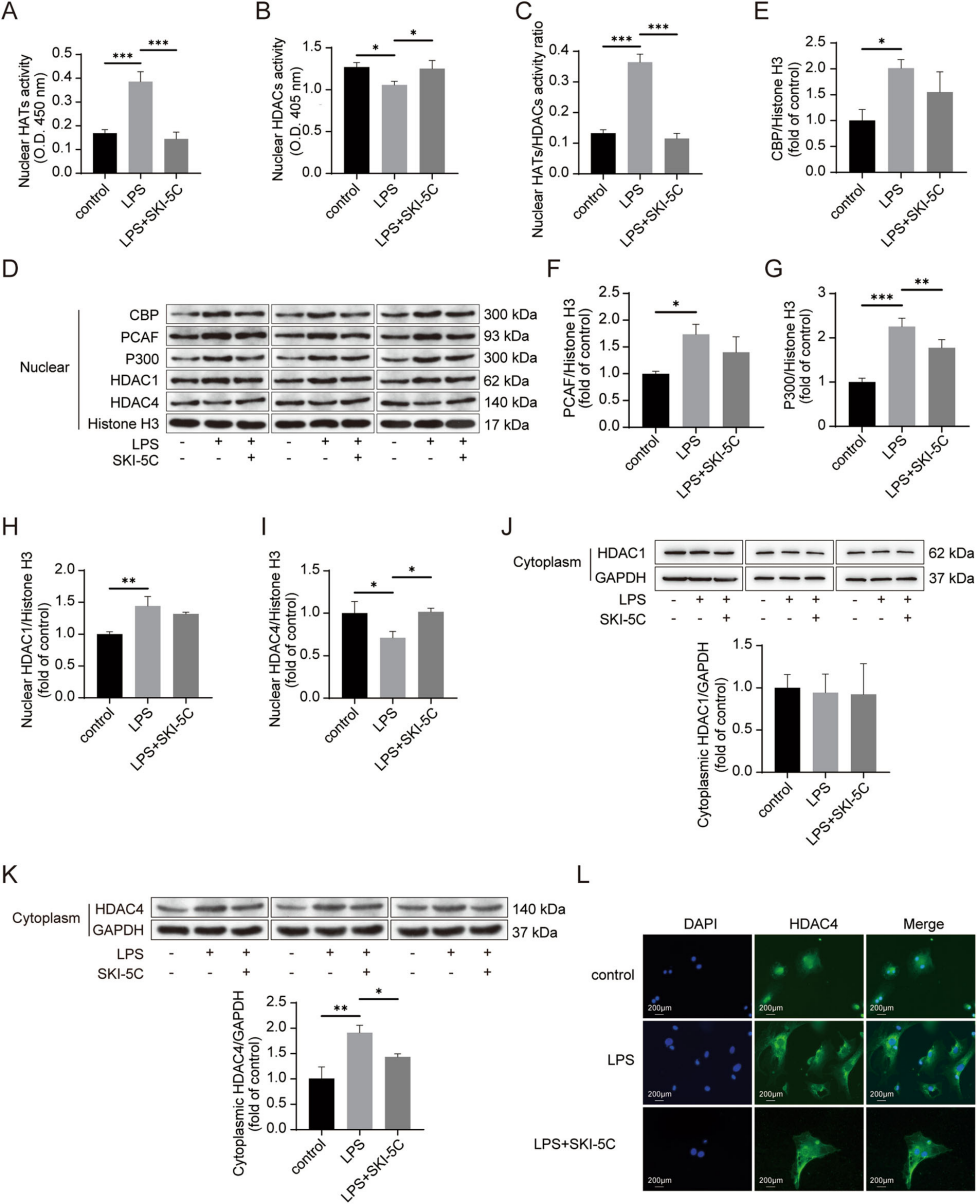
SphK1 inhibition suppresses p300 expression and HDAC4 translocation. RAW264.7 cells were pretreated with SKI-5C (10 μM for 24 h) and then stimulated with LPS (1 μg/ml for 16 h). **A** Colorimetric assay of nuclear HATs activity. **B** Colorimetric assay of HDACs activity. **C** Ratios of nuclear HATs to HDACs activity. **D** Immunoblot analysis of the expression of nuclear CBP, PCAF, p300, HDAC1, and HDAC4. **E**–**I** Quantitative analysis of the expression of nuclear CBP (**E**), PCAF (**F**), p300 (**G**), HDAC1 (**H**), and HDAC4 (**I**). Data represent three independent experiments. **J** Immunoblot analysis of cytoplasmic HDAC1 expression. Data represent three independent experiments. **K** Immunoblot analysis of cytoplasm HDAC4 expression. The data represent three independent experiments. **L** Kupffer cells were pretreated with SKI-5C (10 μM for 24 h) and then stimulated with LPS (1 μg/ml for 16 h). HDAC4 intracellular translocation in Kupffer cells was analyzed by immunofluorescence assay. Data were expressed as the mean ± standard deviation. *P < 0.05, **P < 0.01, ***P < 0.001. HDAC4, nuclear histone deacetyltransferase 4; p300, E1A-associated protein p300; HATs, nuclear histone acetyltransferases; HDACs, nuclear histone deacetyltransferases; CBP, CREB-binding protein; PCAF, p300/CBP-associated factor; and HDAC1, nuclear histone deacetyltransferase 1.

Several studies reported that HMGB1 acetylation was controlled by HATs (CBP, PCAF, and p300)^19–21^ and HDACs (HDAC1 and HDAC4)^22^. HATs activity was increased, whereas HDACs activity was attenuated after LPS treatment of RAW264.7 cells (Fig. 3A, B). These changes were reversed by the SphK1 inhibitor added in vitro (Fig. 3A, B). Compared with the LPS group, the diminished ratio of HATs to HDACs activity was evident in the LPS+ SKI-5C group (Fig. 3C, *P* < 0.001). The protein expression of HATs (CBP, PCAF, and p300) was enhanced by LPS stimulation in vitro (Fig. 3D–G). Decreased expression of p300 was found in the LPS+ SKI-5C group (Fig. 3D, G, *P* < 0.01, compared with the LPS group). In comparison with the LPS group, the protein expression of CBP and PCAF was also reduced by SKI-5C addition in vitro, but the difference was not significant (Fig. 3D–F). SphK1 inhibition did not change the extent of HDAC1 translocation in vitro (Fig. 3D, H, J). The function of HDAC4 is regulated by subcellular localization^23^. In the present study, LPS stimulation resulted in the translocation of HDAC4 from the nucleus to the cytoplasm in vitro (Fig. 3D, I, K). However, HDAC4 intracellular translocation was interrupted by SphK1 inhibition in vitro (Fig. 3D, I, K). Furthermore, immunofluorescence assays confirmed the inhibitory effect of SKI-5C on the HDAC4 intracellular shuttle in Kupffer cells (Fig. 3L).

### SphK1 might directly interact with CaMKII-δ to regulate the phosphorylation of HDAC4

**Fig. 4:**
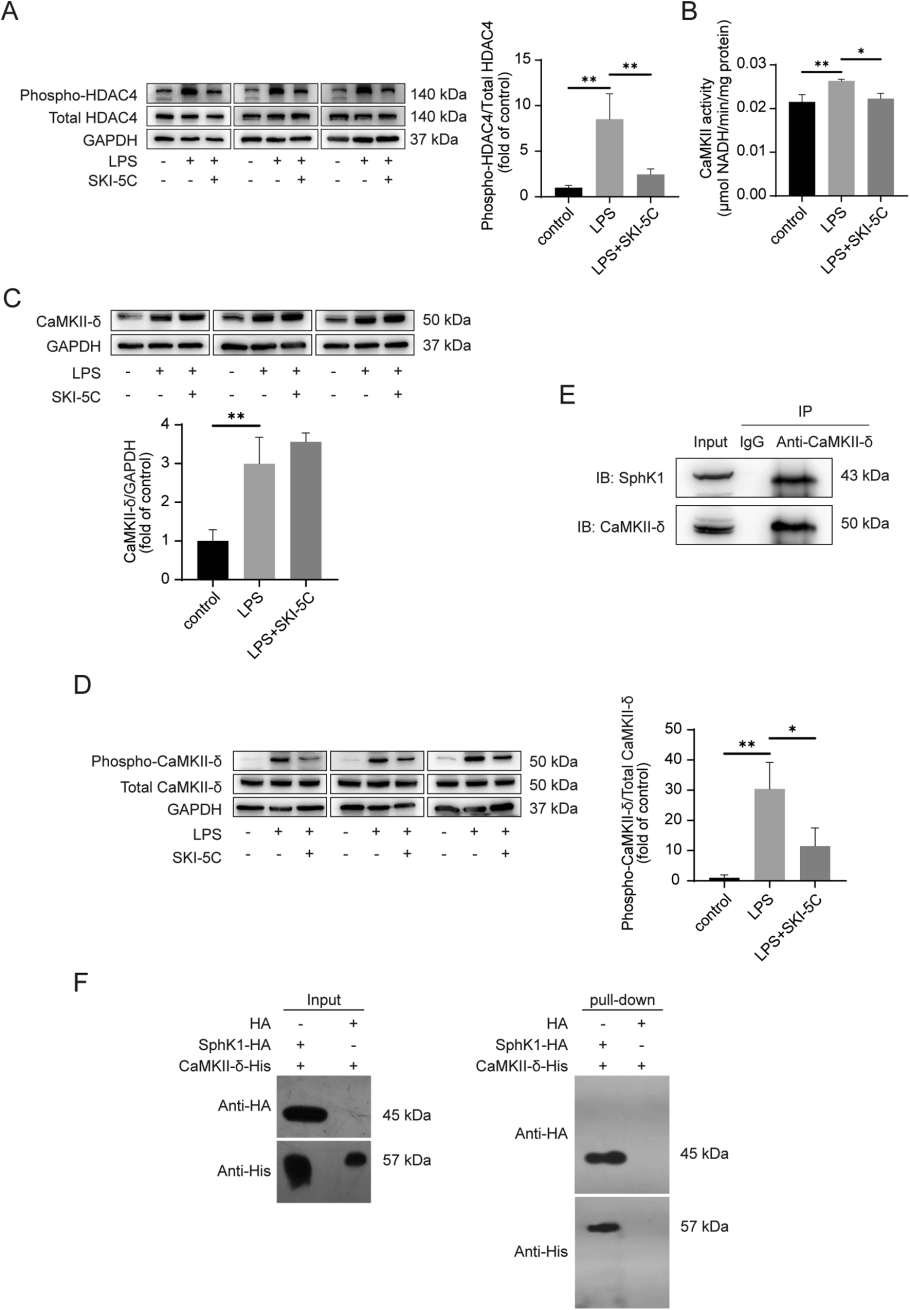
SphK1 directly interacts with CaMKII-δ to regulate the phosphorylation of HDAC4. RAW264.7 cells were pretreated with SKI-5C (10 μM for 24 h) and then stimulated with LPS (1 μg/ml for 16 h). **A** Immunoblot analysis of Phospho-HDAC4. Data represent three independent experiments. **B** Colorimetric assay of CaMKII activity. **C** Immunoblot analysis of CaMKII-δ expression. Data represent three independent experiments. **D** Immunoblot analysis of phospho-CaMKII-δ. Data represent three independent experiments. **E** Co-immunoprecipitation analysis of the interaction between SphK1 and CaMKII-δ in LPS-stimulated RAW264.7 cells. **F** Pull-down analysis of HA, HA-SphK1, and His-CaMKII-δ proteins. Data are expressed as the mean ± standard deviation. *P < 0.05, **P < 0.01, ***P < 0.001. CaMKII-δ, calcium/calmodulin protein kinase II-δ.

**Fig. 5:**
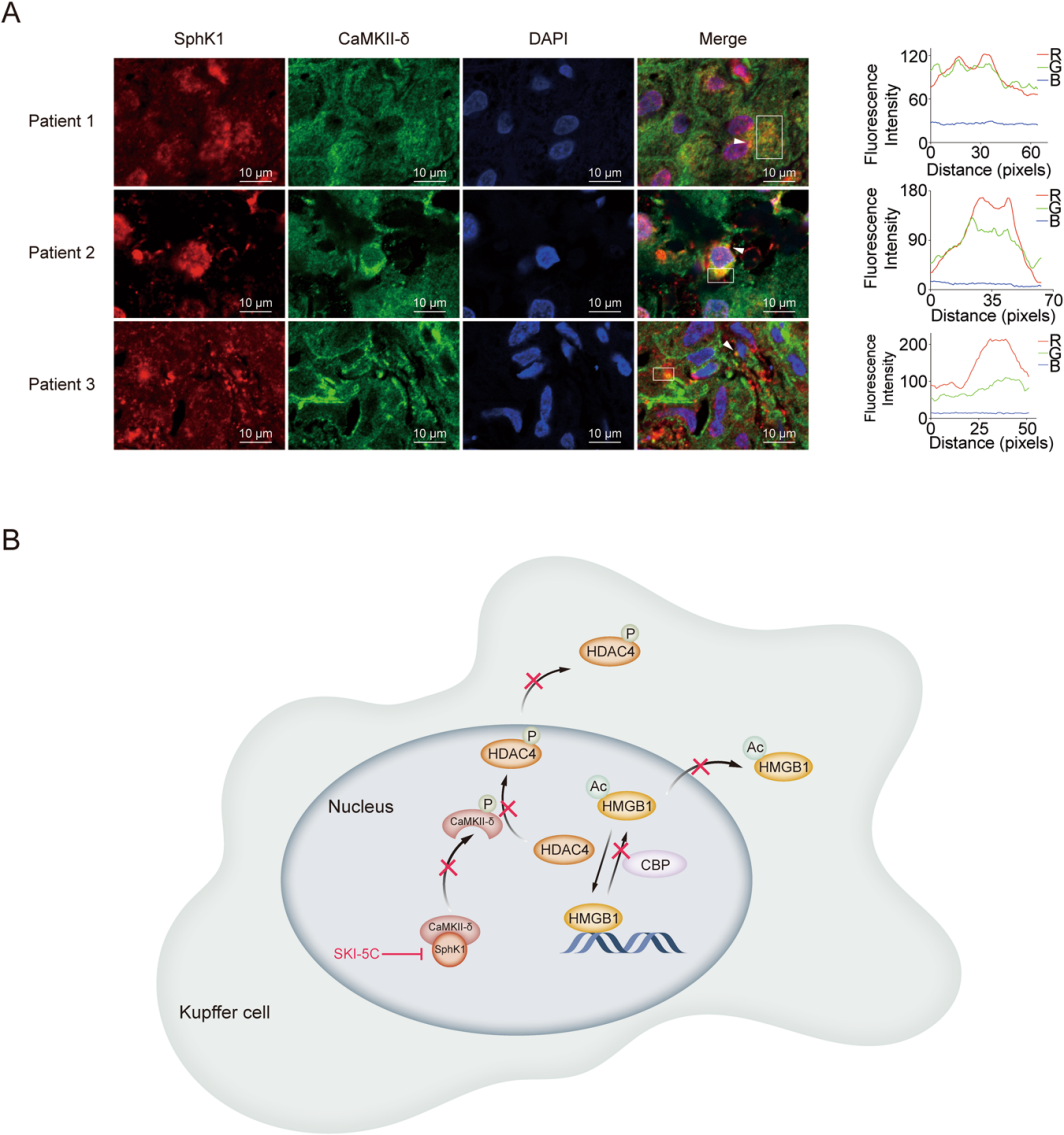
SphK1 colocalizes with CaMKII-δ in the liver of patients with sepsis-associated liver injury. **A** Colocalization analysis of SphK1 and CaMKII-δ in the livers of patients with sepsis-associated liver injury by immunofluorescence assay (*n* = 3). **B** A proposed model shows that SphK1 modulates HMGB1 translocation by directly interacting with CaMKII-δ in sepsis-associated liver injury. SphK1 can directly interact with CaMKII-δ in Kupffer cells. SphK1 inhibition may reduce the phosphorylation of CaMKII-δ after LPS (1 μg/ml) stimulation, which results in a decline in HDAC4 phosphorylation. HDAC4 intracellular translocation is then suppressed. HMGB1 acetylation is controlled by HATs and HDACs. SphK1 inhibition causes HDAC4 accumulation and decreased CBP expression in the nucleus after LPS (1 μg/ml) stimulation, which attenuates HMGB1 intracellular translocation. Data are expressed as the means ± standard deviation. *P < 0.05, **P < 0.01, ***P < 0.001. CaMKII-δ, calcium/calmodulin protein kinase II-δ; HATs, nuclear histone acetyltransferases; HDACs, nuclear histone deacetyltransferases; HDAC4, nuclear histone deacetyltransferase 4; and CBP, CREB-binding protein.

Phosphorylation is the main mechanism for HDAC4 translocation from the nucleus to the cytoplasm^23^. As shown in Fig. 4A, LPS stimulation enhanced the level of phospho-HDAC4 in RAW264.7 cells, while SKI-5C downregulated it. HDAC4 phosphorylation is known to be modulated by calcium/calmodulin-dependent kinase II-δ (CaMKII-δ)^24,25^. Figure 4B indicates that LPS-activated CaMKII activity was significantly decreased by SKI-5C in vitro (*P* < 0.05, compared with the LPS group). However, the high expression of CaMKII-δ stimulated by LPS was not changed by SKI-5C in vitro (Fig. 4C). Autophosphorylation occurs when the CaMKII-δ enzyme is continuously activated^26^. In our study, the phosphorylation of CaMKII-δ induced by LPS was hindered by SKI-5C in vitro (Fig. 4D, *P* < 0.05, compared with the LPS group). Notably, the in vitro co-IP and pull-down assays revealed that SphK1 might directly interact with CaMKII-δ (Fig. 4E, F). Then we confirmed the colocalization of SphK1 and CaMKII-δ in the livers of patients with sepsis-associated liver injury by immunofluorescence assay (Fig. 5A).

## Discussion

In this study, we verified that Kupffer cells were the major cells secreting HMGB1 in the liver. HMGB1 expression, intracellular translocation, and acetylation were suppressed by SphK1 inhibition in sepsis-associated liver injury. HDAC4 intracellular shift and p300 expression were also suppressed by SphK1 inhibition. Additionally, SphK1 can directly interact with CaMKII-δ. Then, we confirmed the colocalization of SphK1 and CaMKII-δ in liver tissues of sepsis-associated liver injury patients. These findings imply that SphK1 controls HMGB1 translocation by directly interacting with CaMKII-δ, which might contribute to the pathogenesis of sepsis-associated liver injury.

Immune cells such as mature dendritic cells, natural killer cells, and macrophages have been reported to actively secrete HMGB1^27,28^. Kupffer cells, the resident macrophages in the liver, have been demonstrated to release HMGB1 after LPS treatment^29^. Hepatocytes have also been shown to release HMGB1 in ALF^5,22,30^. However, which cells in the liver are predominantly secreting HMGB1 during sepsis-associated liver injury was unknown. In this study, the HMGB1 concentration in the supernatant of primary rat Kupffer cells in culture was dramatically higher than that of primary rat hepatocytes after the cells were treated with LPS in vitro. GdCl3, a widely used Kupffer cell depletion reagent, can inactivate the reticulo-endothelial system macrophages and suppress Kupffer cell phagocytosis^31^. We observed that serum HMGB1 was strikingly decreased by Kupffer cell depletion. These results suggest that Kupffer cells may be the cells primarily secreting HMGB1 in the liver during sepsis-associated liver injury.

Previous studies have reported that SphK1 and HMGB1 are activated in sepsis-associated liver injury^7,11^. Therefore, we explored the association between SphK1 and HMGB1. In this study, Sphk1 inhibition diminished HMGB1 expression, which was in accordance with a previous study^32^. The underlying mechanism may be that SphK1 plays a key role in proinflammatory cytokines production through NF-κB activation^33^. Before active release, HMGB1 needs to be shuttled from the nucleus to the cytoplasm^5,34^. We demonstrated that LPS-mediated subcellular localization of HMGB1 was blocked by SphK1 inhibition during sepsis-associated liver injury. The mechanism might be associated with the direct interaction of SphK1 and CaMKII-δ in Kupffer cells. CaMKII-δ may undergo autophosphorylation when it is continuously activated^25,26^. CaMKII-δ has been shown to control the phosphorylation of HDAC4, which accelerates nuclear export and inhibits nuclear import of HDAC4^23,24,35^. HDAC4 can shuttle from the nucleus to the cytoplasm upon stimulation, which is the main mechanism regulating enzyme activity^22,23,36^. HDAC4 may enhance the deacetylated form of HMGB1, which diminishes HMGB1 translocation^19,22^. In agreement with a previous study, we also observed that the activity and phosphorylation of CaMKII were activated by LPS^37,38^. However, these changes were completely altered by SphK1 inhibition. In our study, SphK1 inhibition reduced the activity and phosphorylation of CaMKII-δ after LPS stimulation, which resulted in a decline in HDAC4 phosphorylation. Intracellular HDAC4 translocation was then suppressed. The inhibition of intracellular HDAC4 shift might attenuate HMGB1 acetylation, which contributes to the decrease in HMGB1 translocation. These findings suggest that the direct interaction between SphK1 and CaMKII-δ regulates the phosphorylation and intracellular shift of HDAC4, which modulates HMGB1 translocation.

Previous studies have reported that HATs (CBP, PCAF, and p300) can acetylate HMGB1, which facilitates the intracellular translocation of HMGB1^20,21,39^. In this study, HATs expression and activity were upregulated by LPS, which is consistent with previous reports^39,40^. However, p300 expression was significantly reduced by SphK1 inhibition. Decreased p300 expression might cause a reduction in the level of HMGB1 acetylation, which inhibits HMGB1 translocation. This finding was consistent with a previous study reporting that the mRNA expression of CBP/p300 was downregulated by SphK1 siRNA in mesothelioma cells^41^. These results suggest that SphK1 inhibition might decrease p300 expression to modulate HMGB1 translocation.

The primary shortcoming of this study is the lack of SphK1-knockout animals. We used only a specific chemical inhibitor. Moreover, further experiments are needed to explore the regions and motifs that mediate the interaction between SphK1 and CaMKII-δ.

In conclusion, this study shows that SphK1 inhibition suppresses HMGB1 intracellular translocation in sepsis-associated liver injury. The underlying mechanism is associated with the direct interaction of SphK1 and CaMKII-δ. These observations reveal that SphK1 regulates HMGB1 translocation to participate in the development of sepsis-associated liver injury.

## Supplementary information

Supplementary Methods

Supplemental figure 1 legend

Supplemental table 1

Supplementary table 2

Supplementary figure 1
