# Supplementary material for: Sphingosine kinase 1 regulates HMGB1 translocation by directly interacting with calcium/calmodulin protein kinase II-δ in sepsis-associated liver injury
Source: Cell Death Dis. 2020 Dec 6;11(12):1037. doi: 10.1038/s41419-020-03255-6 (PMC7719708; doi:10.1038/s41419-020-03255-6)
Supplement: Supplementary file 1 — Supplementary Methods [file 41419_2020_3255_MOESM1_ESM.docx]

**Methods**

**1. Primary Kupffer cell isolation**

The protocol of primary Kupffer cell isolation from SD rats mainly contains three steps: in situ liver perfusion with collagenase digestion, gradient centrifugation, and selective adherence. The perfusion buffer was 50 ml Roswell Park Memorial Institute 1640 (RPMI 1640, Hyclone) containing 0.05 % type IV collagenase. After in situ liver perfusion, the liver homogenate was centrifuged twice at 300 × g (4 ℃) for 5 min to wash out the residual collagenase and tissue fragments. The pellet was kept and the supernatant was discarded. Then the pellet was then centrifuged at 50 × g (4 ℃) for 3 min to separate liver non-parenchymal cells from parenchymal cells. The supernatant was kept and centrifuged at 300 × g (4 ℃) for 5 min. The cell pellet was kept and cultured in Dulbecco’s Modified Eagle Medium (DMEM) supplemented with 10% fetal bovine serum (FBS), 100 U/ml penicillin, and 100 μg/ml streptomycin. After incubation for 2 h, the cells were gently washed with PBS.

**2. Primary hepatocyte isolation**

The procedure of in situ liver perfusion was the same as the step of primary Kupffer cell isolation. The liver homogenate was centrifuged thrice at 50 × g (4 ℃) for 3 min. The cell pellet was seeded on plates pre-coated with rat tail collagen and cultured in William’ s E Medium supplemented with 10 % fetal bovine serum (FBS), 100 U/ml penicillin, and 100 μg/ml streptomycin.

**3.** **Separation of cytoplasmic and nuclear extracts**

Cytoplasmic and nuclear extracts were separated with a cell fractionation kit (CST). About 5 x 10^6^ cells were trypsinized. Cells were spun down at 350 x g for 5 min. Cell pellets were resuspended in 500 μl of cytoplasm isolation buffer. The samples were vortexed for 5 seconds and incubated on ice for 5 min. Then samples were then centrifuged for 5 min at 500 x g. The supernatant was the cytoplasmic fraction. The rest of the pellets were resuspended in 500 μl of membrane isolation buffer. The samples were vortexed for 15 seconds and incubated on ice for 5 min. Then samples were then centrifuged for 5 min at 8,000 x g. The supernatant was the membrane and organelle fraction. The rest of the pellets were resuspended in 250 μl of nucleus isolation buffer. The samples were sonicated for 5 seconds at 15% power 3 times. This was the nuclear fraction.

**4. Immunoprecipitation (IP) and co-****immunoprecipitation (co-IP)**

RIPA buffer was added to each plate. The samples were sonicated on ice three times for 5 seconds each. Then, the samples were centrifuged at 14,000 X g for 10 min at 4 °C. The supernatant was saved. The concentration of samples was detected with a BCA kit. A starting concentration between 250 μg/ml - 1.0 mg/ml is recommended. 20 μl of bead slurry was transferred to a tube. The tube was placed in a magnetic separation rack for 10 seconds. The magnetic bead pellets were washed twice with 500 μl of 1X cell lysis buffer. 200 μl cell lysates were added to 20 μl of pre-washed magnetic beads. The mixture was incubated with rotation for 20 min at room temperature. The beads were separated from the lysate with a magnetic separation rack. The pre-cleared lysate was transferred to a clean tube. The primary antibody was added to the pre-cleared lysate. The mixture was incubated with rotation overnight at 4 °C to form the immunocomplex. Pre-washed magnetic beads were added to the immunocomplex solution. Then, the mixture was incubated with rotation for 20 minutes at room temperature. The beads were precipitated with a magnetic separation rack. The bead pellet was washed five times with 500 μl of 1X cell lysis buffer and resuspended with 20-40 μl 3X SDS sample buffer. The sample was heated at 95-100 °C for 5 min. The beads were separated with a magnetic separation rack. The samples were analyzed by immunoblotting.

**5. Immunoblotting**

The samples were prepared with RIPA buffer. The cell lysates were sonicated for 5 seconds at 15% power 3 times and heated at 95-100 °C for 5 min. The samples were separated by SDS-PAGE gel electrophoresis. The proteins were transferred to polyvinylidene difluoride (PVDF) membranes. The membrane was blocked with 1X TBST with 5% nonfat dry milk for 1 hour at room temperature and washed three times for 5 min each with 1X TBST. Then, the membrane was then incubated with the primary antibody overnight at 4 °C. After three rinses with 1X TBST, the membrane was incubated with a secondary antibody. Images were obtained with a chemiluminescence immunoassay analyzer.

**6. Pull-down assay**

HA-SphK1 fusion protein cloned into the pGEX4T-1 vector and His-CaMKII-δ fusion protein cloned into the pCzn1 vector were transformed into BL21 Escherichia coli. A single colony was transferred into 3 ml of Luria broth (LB) and 50 μg/ml ampicillin. The bacterial was cultured overnight at 37°C. The 3ml bacterial solution was transferred into 30 ml of LB and 50 μg/ml ampicillin. The fusion proteins were expressed at an OD600 of approximately 0.8 with 0.5 mM IPTG for 8 h at 11-28 °C. The bacteria pellet was sonicated three times. The fusion proteins were purified with Nickel column purification. HA-tagged SphK1 and His-tagged CaMKII-δ were added to HA resin. The mixture was rotated at 4 °C overnight. After incubation, the mixture was centrifuged at 1,250 X g for 5 min. The supernatant was discarded. The elution buffer was added to the mixture. After incubation for 20 min at 4 °C, the mixture was centrifuged at 1,250 X g for 2 min. After the election, the protein samples were separated and detected by immunoblotting.

**7. Histone acetyltransferases (HATs) activity assay**

The HATs activity of the nuclear extract was detected by colorimetric assay kits (BioVision). The samples (50 μg of nuclear extract) were prepared in 40 μl water (final volume) for each assay in a 96-well plate. For each well, prepare a total 68 μl Assay Mix containing: 50 μl 2X HAT Assay Buffer, 5 μl HAT Substrate I, 5 μl HAT Substrate II, and 8 μl NADH Generating Enzyme. The plate was incubated at 37 °C for 4 hours. Absorbance at 440 nm was read using a microplate reader.

**8. Histone deacetyltransferases (HDACs) activity assay**

The HDACs activity of the nuclear extract was detected by colorimetric assay kits (BioVision). The samples (50 μg of nuclear extract) were prepared in 85 μl (final volume). 10 μl of the 10X HDAC Assay Buffer and 5 μl of the HDAC colorimetric substrate was added to each well. After incubation at 37 °C for 4 hours, 10 μl of Lysine Developer was added to stop the reaction. After incubation at 37 °C for 30 min, absorbance at 405 nm was read using a microplate reader.

**9. Calcium/Calmodulin Protein Kinase II (CaMKII) activity assay**

CaMKII activity was measured using a commercial assay kit (Genmed). 60 μl of Reagent A and 50 μg of the sample were pipetted into each well. The plate was incubated for 10 min at 30 °C. Then, 10 μl of Reagent B, C, and D were pipetted into each well. Absorbance at 340 nm was read every 5 min using a microplate reader. CaMKII activity was presented as μmol NADPH/min/mg protein.

**10. ELISA**

HMGB1 in serum, cell lysis, and supernatant was measured with a commercial ELISA kit (IBL International). Cell lysis was prepared with PathScan® Sandwich ELISA Lysis buffer (CST). For the lysis of adherent cells, lysis buffer was added to the dishes. The plate was incubated on ice for 2 min. The extract was centrifuged at 14,000 X g for 5 min. The sample was added to the microtiter plate. After incubation for 24 hours at 37 °C, the plate was washed 5 times with 400 μl diluted wash buffer. 100 μl of enzyme conjugate was pipetted into each well. The plate was incubated for 2 hours at 25 °C. And then, the plate was washed 5 times with 400 μl diluted wash buffer. 100 μl of the color solution was pipetted into each well. The plate was incubated for 30 min at room temperature (18-25°C). The stop solution was pipetted into each well. Absorbance at 450 nm was measured using a microplate reader.

**11.** **Immunofluorescence**

For human liver tissues, the samples were immersed in 10% formalin for 18-24h. The tissues were then embedded in paraffin. After the deparaffinization and rehydration step, antigen retrieval was performed by heat retrieval. Cells were grown and stained in chamber slides. The slide was fixed with 4% formaldehyde for 15 min at room temperature and rinsed three times in 1X PBS for 5 min each. The blocking buffer was added to the slide for 60 min. The slide was incubated with the primary antibody overnight at 4 °C. After incubation with the primary antibody, the slide was rinsed three times in 1X PBS for 5 min each. The slide was then incubated with a secondary antibody for 1 hour at room temperature in the dark and rinsed three times in 1X PBS for 5 min each. Images were obtained with a Nikon fluorescence microscope.
