# Supplementary material for: Sphingosine kinase 1 regulates HMGB1 translocation by directly interacting with calcium/calmodulin protein kinase II-δ in sepsis-associated liver injury
Source: Cell Death Dis. 2020 Dec 6;11(12):1037. doi: 10.1038/s41419-020-03255-6 (PMC7719708; doi:10.1038/s41419-020-03255-6)
Supplement: Supplementary file 2 — Supplemental figure 1 legend [file 41419_2020_3255_MOESM2_ESM.docx]

**Supplemental figure legend**

**Supplemental figure 1. Photomicrographs of Kupffer cells and hepatocytes in culture.** Primary Kupffer cells and hepatocytes were isolated from SD rats. Scale bar: 50 μm.
