# Supplementary material for: Sphingosine kinase 1 regulates HMGB1 translocation by directly interacting with calcium/calmodulin protein kinase II-δ in sepsis-associated liver injury
Source: Cell Death Dis. 2020 Dec 6;11(12):1037. doi: 10.1038/s41419-020-03255-6 (PMC7719708; doi:10.1038/s41419-020-03255-6)
Supplement: Supplementary file 3 — Supplemental table 1 [file 41419_2020_3255_MOESM3_ESM.docx]

| **Supplementary Table 1 Antibody information.** | | |
| --- | --- | --- |
| **Antibodies** | **Manufacturers** | **Catalog Number** |
| Anti-CD68 | Thermo Scientific | MA5-13324 |
| Anti-phospho-HDAC4 | Thermo Scientific | PA5-104929 |
| Anti- Calcium/Calmodulin Protein Kinase II-δ (CaMKII-δ) | Thermo Scientific | PA5-22168 |
| And anti-phospho- CaMKII-δ | Thermo Scientific | MA5-37833 |
| Anti-SphK1 | Abcam | Ab71700 |
| anti-HMGB1 (ChIP Grade) | Abcam | Ab18256 |
| Anti-HMGB1 | Cell Signaling Technology | 6839S |
| Anti-GAPDH | Cell Signaling Technology | 2118S |
| Anti-Lamin B1 | Cell Signaling Technology | 13435S |
| Anti-Histone H3 | Cell Signaling Technology | 4499S |
| Anti-E1A-associated protein p300 (p300) | Cell Signaling Technology | 86377S |
| Anti-CREB-binding protein (CBP) | Cell Signaling Technology | 7389S |
| Anti- p300/CBP-associated factor (PCAF) | Cell Signaling Technology | 3378S |
| Anti-histone deacetylase (HDAC) 1 | Cell Signaling Technology | 34589S |
| Anti-HDAC4 | Cell Signaling Technology | 7628S |
| Anti-Acetylated-Lysine | Cell Signaling Technology | 9441S |
| Anti-rabbit IgG, HRP-linked Antibody | Cell Signaling Technology | 7074S |
| Anti-HMGB1 (Acetyl-Lys12) | Aviva Systems Biology | OASG03545 |
| Anti-HA | Zoonbio Technology | — |
| Anti-His | Zoonbio Technology | — |
