# Supplementary material for: Sphingosine kinase 1 regulates HMGB1 translocation by directly interacting with calcium/calmodulin protein kinase II-δ in sepsis-associated liver injury
Source: Cell Death Dis. 2020 Dec 6;11(12):1037. doi: 10.1038/s41419-020-03255-6 (PMC7719708; doi:10.1038/s41419-020-03255-6)
Supplement: Supplementary file 4 — Supplementary table 2 [file 41419_2020_3255_MOESM4_ESM.docx]

| **Supplementary Table 2 Characteristics of the patients.** | | | | |
| --- | --- | --- | --- | --- |
| **Characteristics** | **Patient 1** | **Patient 2** | **Patient 3** | **reference interval** |
| Age/Sex | 76/M | 32/M | 63/F | ‐ |
| Diagnosis | intestinal fistula; sepsis-associated liver damage | intra-abdominal infection; sepsis-associated liver damage | intestinal fistula; sepsis-associated liver damage | ‐ |
| APACHE II score | 22 | 10 | 9 | ‐ |
| SOFA score | 10 | 8 | 11 | ‐ |
| CRP (mg/L) | 92.2 | 63.9 | 90.05 | 0-5 |
| PCT (ng/ml) | 4.27 | 10.78 | 1.2 | 0-0.1 |
| WBC (×10^9^/L) | 3.3 | 15 | 12.3 | 3.5-9.5 |
| RBC (×10^12^/L), | 2.25 | 3.37 | 2.8 | 3.8-5.1 |
| Hb (g/L) | 66 | 113 | 82 | 115-150 |
| PLT (×10^9^/L) | 120 | 168 | 259 | 125-350 |
| PT (s) | 14.2 | 13 | 17.7 | 9.8-12.7 |
| APTT (s) | 47 | 35.7 | 54.9 | 22-36 |
| INR | 1.25 | 1.4 | 1.59 | 0.82-1.15 |
| Albumin (g/L) | 35 | 30 | 31 | 32-48 |
| ALT (U/L) | 18 | 27 | 11 | 10-49 |
| AST (U/L) | 25 | 36 | 18 | 0-34 |
| Total Bilirubin (umol/L) | 202 | 81 | 37 | 5-21 |
| Creatinine (umol/L) | 69 | 71 | 34 | 40-66 |
| BUN (mmol/L) | 9.1 | 12.4 | 6 | 2.5-6.4 |
| Outcome | dead | well | well | - |
| Abbreviations: APACHE, Acute Physiology and Chronic Health Evaluation; SOFA, Sequential Organ Failure Assessment; CRP, C-reaction protein; PCT, procalcitonin; WBC, white blood cell; RBC, red blood cell; Hb, hemoglobin; PLT, platelet; PT, prothrombin time; APTT, activated partial thromboplastin time; INR, International Normalized Ratio; ALT, Alanine aminotransferase; AST, Aspartate amino Transferase; BUN, blood urea nitrogen | | | | |
