# Supplementary figures and images for: Sphingosine kinase 1 regulates HMGB1 translocation by directly interacting with calcium/calmodulin protein kinase II-δ in sepsis-associated liver injury
Source: Cell Death Dis. 2020 Dec 6;11(12):1037. doi: 10.1038/s41419-020-03255-6 (PMC7719708; doi:10.1038/s41419-020-03255-6)

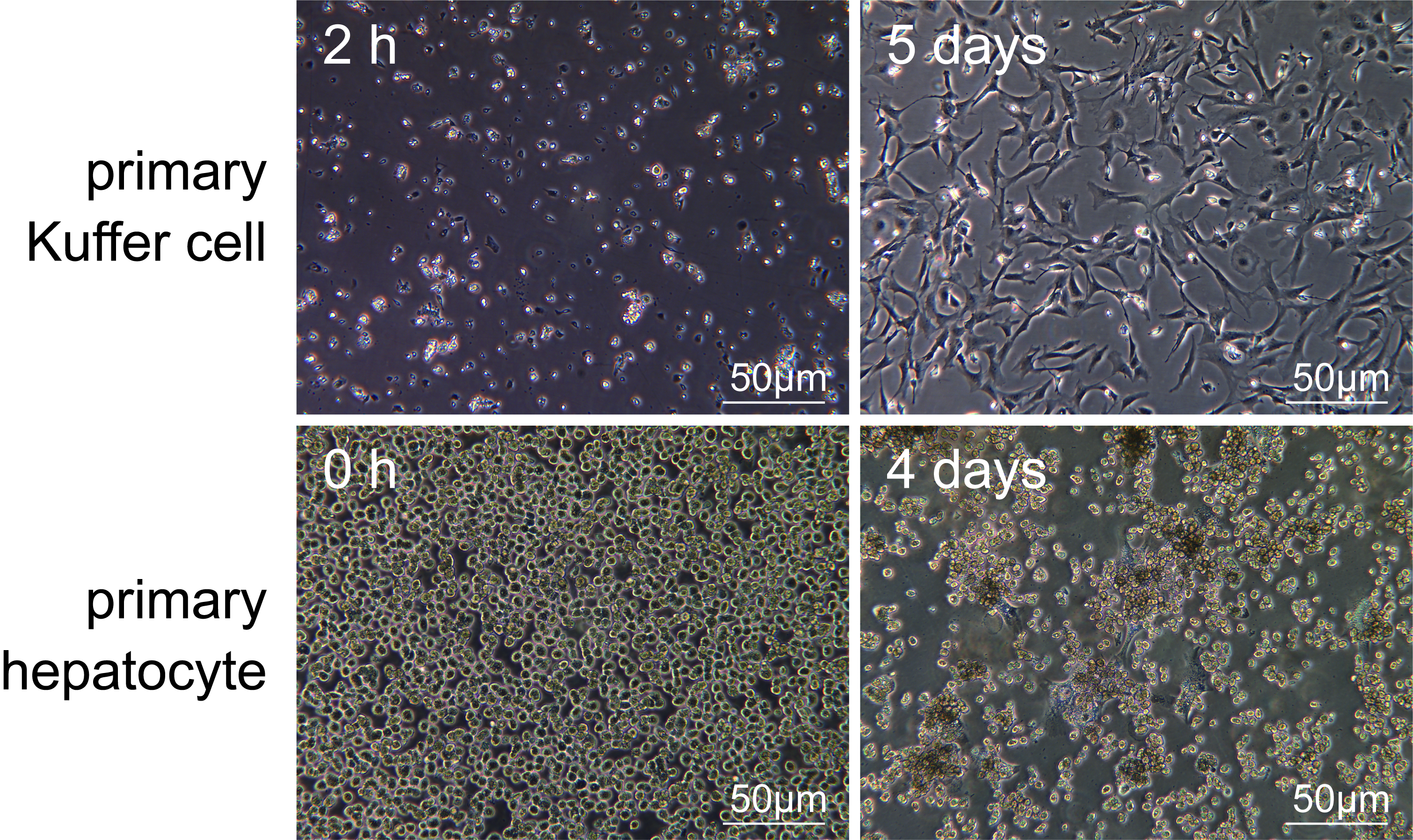

Supplement: Supplementary file 5 — Supplementary figure 1 [file 41419_2020_3255_MOESM5_ESM.tif]
